# Connexin43 Carboxyl-Terminal Domain Directly Interacts with β-Catenin

**DOI:** 10.3390/ijms19061562

**Published:** 2018-05-24

**Authors:** Gaelle Spagnol, Andrew J. Trease, Li Zheng, Mirtha Gutierrez, Ishika Basu, Cleofes Sarmiento, Gabriella Moore, Matthew Cervantes, Paul L. Sorgen

**Affiliations:** Department of Biochemistry and Molecular Biology, University of Nebraska Medical Center, Omaha, NE 68198, USA; gspagnol@unmc.edu (G.S.); andrew.trease@unmc.edu (A.J.T.); li.zheng@unmc.edu (L.Z.); MGutierrez4413@CSM.edu (M.G.); ishika.basu@unmc.edu (I.B.); cleofes.sarmiento@unmc.edu (C.S.); gabriella.moore@unmc.edu (G.M.); Matthew.R.Cervantes.9@nd.edu (M.C.)

**Keywords:** Cx43, β-catenin, phosphorylation

## Abstract

Activation of Wnt signaling induces Connexin43 (Cx43) expression via the transcriptional activity of β-catenin, and results in the enhanced accumulation of the Cx43 protein and the formation of gap junction channels. In response to Wnt signaling, β-catenin co-localizes with the Cx43 protein itself as part of a complex at the gap junction plaque. Work from several labs have also shown indirect evidence of this interaction via reciprocal co-immunoprecipitation. Our goal for the current study was to identify whether β-catenin directly interacts with Cx43, and if so, the location of that direct interaction. Identifying residues involved in direct protein–protein interaction is of importance when they are correlated to the phosphorylation of Cx43, as phosphorylation can modify the binding affinities of Cx43 regulatory protein partners. Therefore, combining the location of a protein partner interaction on Cx43 along with the phosphorylation pattern under different homeostatic and pathological conditions will be crucial information for any potential therapeutic intervention. Here, we identified that β-catenin directly interacts with the Cx43 carboxyl-terminal domain, and that this interaction would be inhibited by the Src phosphorylation of Cx43CT residues Y265 and Y313.

## 1. Introduction

Gap junctions are intercellular channels that permit the passage of ions, small metabolites, and signaling molecules between neighboring cells [1]. They are important in a number of physiological processes, including cellular development, growth, and differentiation. In the heart, gap junctions mediate the propagation of cardiac action potentials and the maintenance of a regular beating rhythm [2]. Dysfunctional intercellular communication via gap junctions has been implicated in causing many human diseases [3]. Gap junctions are formed by the apposition of connexons from adjacent cells, where six connexin proteins form each connexon. Although the 21-connexin isoforms (e.g., 43-kDa isoform, Cx43) share significant sequence homology, differences in the amino acid sequence occur in the cytoplasmic loop and carboxyl terminal (CT) domains.

The CT domain is involved in regulating the trafficking of connexons to and from the plasma membrane, as well as the level of gap junction intercellular communication via a number of post-translational modifications and interactions with protein partners [4,5,6,7,8,9,10,11]. The CT domain is predominately unstructured (i.e., intrinsically disordered), making it an ideal substrate for the regulation of intercellular signaling by facilitating both high specificity and low affinity interaction with many different binding partners to allow the rapid feedback to cytoplasmic signals [12,13,14,15]. Over 20 protein partners have been identified to directly interact with Cx43, which can be categorized according to those that can promote or inhibit intercellular communication (for review see [16,17]). For example, connexin localization and stability at the gap junction plaques are strongly determined by interaction with cytoskeletal-associated proteins; these interactions are modulated by specific phosphorylation events [18,19]. The demonstration that Src phosphorylates Y247 and Y265 of Cx43 [20,21] enabled subsequent findings that pY247 inhibits the Cx43 interaction with β-tubulin [22] and that pY265 inhibits the Cx43 interaction with drebrin [23]. At the gap junction plaque, inhibiting the β-tubulin interaction could be a mechanism involved in the disassembly process. If this inhibition occurs after connexon formation in the trans-Golgi network, trafficking may be re-routed for degradation or to the lateral membrane [24]. The depletion of drebrin results in impaired gap junction intercellular communication, also by targeting Cx43 for degradation [25]. Further, while Cx43CT phosphorylation by Src does not inhibit Zonula occludens-1 (ZO-1) binding, active Src directly competes with Cx43 to bind ZO-1 [26]. Studies from the Gourdie and Lampe labs suggest that interaction with ZO-1 transitions Cx43 from the non-junctional membrane into the gap junction plaque, and that Src inhibits this process [27,28]. Thus, even though our knowledge is no doubt incomplete, it is clear that for Cx43, there exists a network of integrated processes involving phosphorylations and binding partners that control junctional Cx43 [19]. Another protein that has been identified to modulate both Cx43 expression and gap junction intercellular communication is β-catenin [29].

β-catenin is a critical protein in the canonical Wnt signaling transduction cascade. β-catenin (781 amino acids) consists of a well-structured central region made up of 12 armadillo repeats that are flanked by intrinsically disordered N-terminal and C-terminal domains [30,31]. β-catenin is a multi-functional protein whose activity depends on its subcellular localization. β-catenin at the plasma membrane is a component of cell adhesion junctions, while cytosolic accumulation leads to increased nuclear localization and transcriptional activity [32]. The activation of Wnt signaling induces Cx43 expression via the transcriptional activity of β-catenin, which leads to the increased formation of gap junction channels [29,33,34,35,36,37,38]. In response to Wnt signaling, β-catenin co-localizes with the Cx43 protein itself as part of a complex at the gap junction plaque [29]. Unfortunately, none of these studies was able to determine whether the β-catenin and Cx43 interaction is direct, or instead requires other protein partners.

## 2. Results

### 2.1. β-Catenin CT Domain Directly Interacts with the Cx43CT

**Figure 1:**
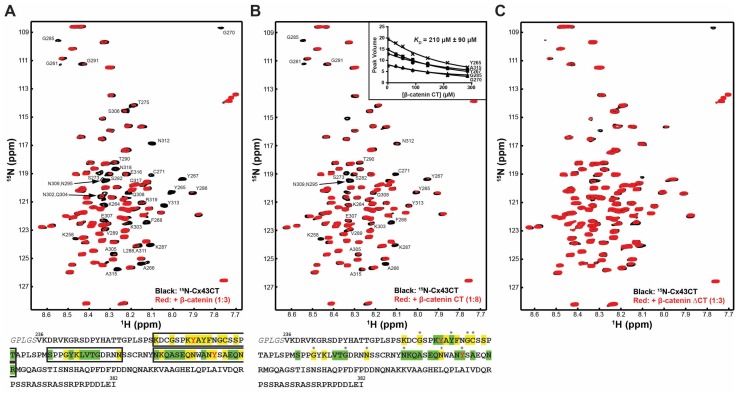
Nuclear magnetic resonance spectra showing the direct interaction between the Cx43CT and β-catenin. ^15^N- heteronuclear single quantum coherence (HSQC) spectra of Cx43CT alone (black) and in the presence of (**A**) full-length β-catenin (red); (**B**) the β-catenin carboxyl-terminal (CT) domain (red); or (**C**) the β-catenin ΔCT domains (red). Molar ratio for each experiment is indicated in the figure. In panel B, provided is a subset of residues used to calculate the *K_D_* of the interaction by fitting their decrease in signal intensity according to β-catenin CT concentration. Below each ^15^N-HSQC spectra is the Cx43CT amino acid sequence. Highlighted are the affected residues (yellow—peaks broadened beyond detection; green—peaks decreased in intensity). Black boxes delimitate the three areas of interaction with β-catenin. Asterisks denote that amino acids that were used to calculate the binding affinity for the β-catenin CT. Two of the residues phosphorylated by Src and affected by β-catenin are also highlighted (red letters).

The domain architecture of β-catenin includes a disordered N-terminal domain (~150 residues; binds α-catenin, glycogen synthase kinase 3β (GSK3β), and is phosphorylated by casein kinase I), a well-structured central armadillo repeat domain (~530 residues; major protein partner binding domain; e.g., binds axin, adenomatous polyposis coli protein, 14-3-3ζ, and E-cadherin), and a disordered C-terminal domain (~100 residues; binds several transcriptional coactivators) [39,40]. To determine whether β-catenin directly interacts with the Cx43CT domain, we performed a ^15^N-heteronuclear single quantum coherence (HSQC) nuclear magnetic resonance (NMR) experiment using purified ^15^N-lableled Cx43CT (V236–I382) and unlabeled full-length β-catenin (Figure 1A, top). Each chemical shift (or peak) in this two-dimensional experiment corresponds to one amide group; thus, the number of peaks should correspond to the number of Cx43CT residues (except proline). We have previously published the ^15^N-HSQC assignment for the Cx43CT domain [41]. These chemical shifts are sensitive to their environment and small changes in structure and/or dynamics, such as those that would occur from a direct protein–protein interaction, can influence the chemical shift (i.e., change the location or broaden beyond detection) of an amino acid. The advantage of using NMR to study direct protein–protein interactions over cellular assays such as immunoprecipitation is its specificity. As only two proteins are present in the solution, any detected interaction is the result of a direct interaction, as opposed to the possibility that both are part of a larger molecular complex (limits of immunoprecipitation). Moreover, because the chemical shifts of the affected amino acids drift or diminish, the specific residues involved in the interaction can be determined. The addition of β-catenin affected a subset of Cx43CT residues (Figure 1A, top). When mapped onto the Cx43CT sequence, they were located within three areas: K259–T275, S282–N295, and N302–R319 (Figure 1A, bottom). The data indicates that the Cx43CT domain and β-catenin directly interact.

**Figure 2:**
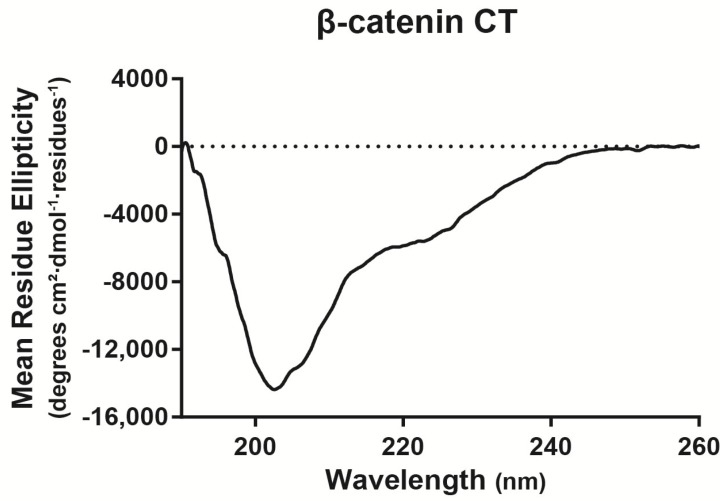
Circular dichroism spectrum showing the secondary structure of the β-catenin CT domain. The spectrum is represented as mean residue ellipticity as a function of wavelength. Data were analyzed using Dichroweb.

**Figure 3:**
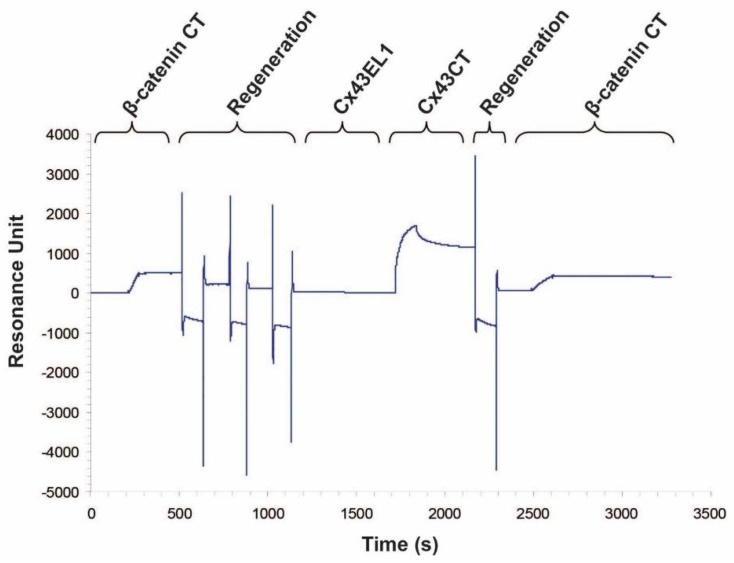
Surface plasmon resonance spectra showing direct interaction between the Cx43CT and β-catenin CT domains. Cx43CT was immobilized onto a CM5 chip by amine coupling (Biacore; GE Healthcare, Uppsala, Sweden) and either β-catenin CT (500 response units), Cx43EL1 (negative control), or Cx43CT (residues S255–I382, positive control) were flown over the chip as indicated on the top of the graph. The chip was regenerated after an interaction was observed. Repeat of an injection of β-catenin CT was performed to confirm the interaction.

To identify the β-catenin domain mediating the direct interaction with Cx43CT, we initially focused on the β-catenin CT domain (S681–L781). The N-terminal domain is the primary locus for Wnt signaling (GSK3β/E3 ubiquitin ligase), and armadillo repeat domains have been well characterized for binding partners involved in cell adhesion. Moreover, most of the binding partners to the C-terminal domain occur in the nucleus, thus potentially leaving the CT domain free to associate with a different set of proteins in the cytoplasm [42]. Upon purification, circular dichroism was used to determine whether the β-catenin CT domain contains any secondary structure (Figure 2). Analysis of the circular dichroism spectrum by Dichroweb (London, UK) determined that the protein is predominately intrinsically disordered with between 18–21% α-helical content [43,44]. The low amount of secondary structure is consistent with the low peak dispersion (<1 ^1^H ppm) that was previously seen in the β-catenin CT domain ^15^N-HSQC [32]. The addition of the unlabeled β-catenin CT domain affected the same subset of ^15^N-Cx43CT residues as full-length β-catenin (Figure 1B, top). These have been highlighted on the Cx43CT sequence (Figure 1B, bottom). Next, a titration of the unlabeled β-catenin CT domain was performed to determine the binding affinity (*K_D_*). The decrease in signal intensity caused by increasing the β-catenin CT domain concentration was fit according to the nonlinear least-square method (Figure 1B, inset). The *K_D_* was determined to be 210 μM (±90 μM). A surface plasmon resonance (SPR) experiment confirmed the NMR results (Figure 3). When the Cx43CT domain was immobilized onto a carboxymethyl-dextran 5 chip, the addition of the β-catenin CT domain resulted in a direct interaction. A peptide to the Cx43 first extracellular loop (EL1, residues G38–R76) served as a negative control, and the Cx43CT domain itself served as a positive control (specific areas of dimerization include M281–N295, R299–Q304, S314–I327, and Q342–A348, [45]). To ensure that the β-catenin CT is the only β-catenin domain interacting with the Cx43CT, we purified a β-catenin construct containing the N-terminal and armadillo repeat domains (β-catenin ΔCT, i.e., deleted the CT domain). The addition of the unlabeled β-catenin ΔCT had no effect on the ^15^N-Cx43CT residues (Figure 1C). Of note, the N-terminal domain of β-catenin has poor expression and degraded during purification, which prevented any attempt to test this domain only. Altogether, the data indicate that the β-catenin CT is the domain that directly interacts with Cx43CT.

### 2.2. Phosphorylation of Y265 and Y313 Inhibits Cx43 Binding with β-Catenin

**Figure 4:**
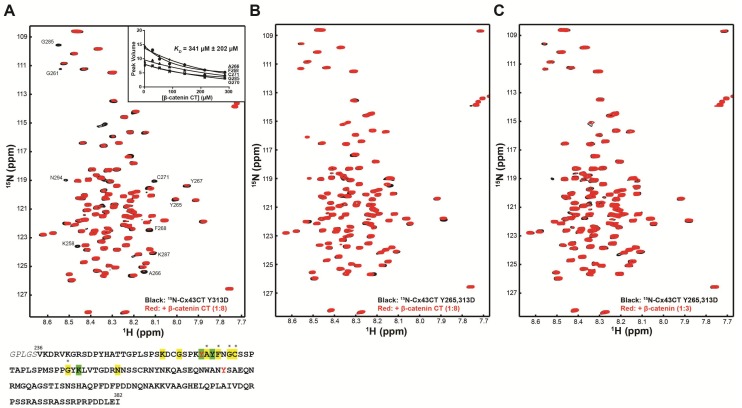
Mimicking phosphorylation of Cx43CT residues Y265 and Y313 inhibits the interaction with the β-catenin CT domain. ^15^N-HSQC spectra of (**A**) Cx43CT Y313D alone (black) and in the presence of the β-catenin CT domain (red) or Y265,313D alone (black), and in the presence of the (**B**) β-catenin CT domain and (**C**) full-length β-catenin (red). The molar ratio for each experiment is indicated in the figure. In panel A, provided is a subset of residues used to calculate the *K_D_* of the interaction by fitting their decrease in signal intensity according to β-catenin CT concentration. Also represented below the ^15^N-HSQC spectra is the Cx43CT amino acid sequence. Highlighted are the affected residues (yellow—peaks broadened beyond detection; green—peaks decreased in intensity). Asterisks denote amino acids used to calculate the binding affinity for the β-catenin CT interaction with the Cx43CT. Tyrosine residues 265 and 313 phosphorylated by Src are indicated in red.

Previous studies have identified that Src phosphorylates Cx43CT residues Y247 and Y265 [21,46]. Additional studies have identified that Src also phosphorylates Cx43CT residues Y313 (Li et al., 2018, Journal of Molecular and Cellular Cardiology, publication under revision; PhosphoSitePlus, [47]). Since the β-catenin CT domain affected Cx43CT residues Y265 and Y313, we addressed whether the phosphorylation of both sites could dissociate β-catenin from Cx43. A number of gap junction studies have determined that an aspartic acid can mimic a phosphate for Cx43 [41,48,49] and responds to the need to purify enough protein for biophysical studies. Therefore, NMR titration experiments were performed using purified soluble ^15^N-labeled Cx43CT single or double phosphomimetics (Y313D or Y265,313D) and different concentrations of either an unlabeled β-catenin CT domain (Figure 4A,B) or full-length β-catenin (Figure 4C)_._ The ^15^N-HSQC spectrum of each control (no β-catenin, black) has been overlaid with spectra when either the β-catenin CT domain or full-length β-catenin (red) were added at a single molar ratio. For the single phosphomimetic construct, among the three areas where the β-catenin CT domain interacted with Cx43CT wild type (WT), binding was completely inhibited for area three (N302–R319), significantly reduced in area two (S282–N295), and mostly preserved in area one (K259–T275). A titration of the unlabeled β-catenin CT domain was performed to determine the *K_D_*. The interaction with β-catenin decreased by approximately two-fold compared to WT (*K_D_* = 341 μM vs. 202 μM) (Figure 4A, inset). When both Cx43CT Y265 and Y313 sites were mutated to mimic Src phosphorylation, the Cx43CT interaction with β-catenin was completely inhibited. These results confirm the direct interaction between Cx43CT and CT portion of β-catenin, and strongly suggest that Src phosphorylation of Cx43CT regulates this interaction.

## 3. Discussion

The Cx43CT domain binds multiple proteins, many of which have been shown to regulate Cx43 function through altering trafficking to the gap junction plaque, opening/closing gap junction channels, disassembly, and degradation (for review see [16,50]). Numerous excellent reviews have summarized the functional significance of these Cx43-interacting proteins [50,51,52,53]. These proteins can be partitioned into those that directly interact with the Cx43CT, and those proteins that can affect all aspects of the Cx43 life cycle, but no current evidence exists that they directly interact with the Cx43CT. One of the proteins in the latter category is β-catenin. Work from several labs has shown indirect evidence of this interaction, including reciprocal co-immunoprecipitation as well as co-localization of Cx43 with β-catenin [29,35]. Additionally, β-catenin segregates in triton insoluble fractions with Cx43 [29]. Our goal here was to identify whether β-catenin directly interacts with Cx43, and the location of that direct interaction.

β-catenin is an intracellular signal transducer in the Wnt signaling pathway that is involved in the regulation and coordination of cell–cell adhesion and gene transcription [54]. Ai et al. first identified that in response to Wnt signaling by the addition of Li^+^, β-catenin interacts with the Cx43 gene *GJA1* (contains three transcription factor 4 (TCF)/lymphoid enhancer binding factor binding sites; [55]) to increase transcription expression [29]. The accumulating Cx43 in the junctional membrane increased neonatal rat cardiomyocyte cell-to-cell coupling and co-localization with β-catenin [29]. Cx43 and β-catenin co-localization also occurs at the intercalated discs of adult rat cardiomyocytes [56]. A similar response to Li^+^ elicited Wnt/β-catenin signaling, which increased Cx43 expression and gap junction intercellular communication in skeletal myoblasts [57]. Additionally, the rapid electrical stimulation of cardiomyocytes had a similar effect as Li^+^, leading to the increased nuclear localization of β-catenin and subsequent Cx43 expression [58]. The suggested sequestering of β-catenin by Cx43 would serve to reduce the transactivation potential of β-catenin [29]. This observation is consistent with the increase in the active form of β-catenin with the knockdown of Cx43 seen in human neural progenitor cells [35], and the decrease in the nuclear localization of β-catenin with Cx43 overexpression in breast adenocarcinoma cell lines [36]. Conversely, Moorer et al. observed in a Cx43CT truncation (K258stop) mouse model that osteoblasts had reduced active β-catenin (along with protein kinase C δ and extracellular signal-regulated kinase 1/2), leading to altered proliferation, differentiation, collagen processing, and organization [34]. While the phenotype observed from loss of the Cx43CT matches that from the complete loss of Cx43 in bone cells, the same is not true in the cardiovascular system [34,59]. This suggests the influence of Cx43 on the activity and cellular localization of β-catenin may be tissue specific [34,59].

Shaw et al. put forth a model for connexin trafficking to the plasma membrane [56]. Cx43 oligomerizes into connexons in the trans-Golgi network [60]. Upon exiting, they use microtubules to travel to adherens junctions, which capture the microtubules allowing for connexon offloading to the plasma membrane [56,61,62,63,64]. Based upon the co-localization of Cx43 and β-catenin at the gap junction plaque, at some point in the trafficking, either to or at the adherens junctions, the interaction occurs. The β-catenin interaction occurs at Cx43 residues K259–T275, S282–N295, and N302–R319. Interestingly, similar residues directly interact with drebrin [23]. A commonality with these proteins is they would both help Cx43 indirectly interact with F-actin (β-catenin indirectly through α-catenin; drebrin directly) and stabilize gap junctions to favor intercellular communication. Conversely, they both cannot interact at the same time. Therefore, the available data would suggest that β-catenin binds first, and then at some point in the maturation of the gap junction plaque, Cx43CT switches to interact with drebrin. Since the phosphorylation of Y265 and Y313 also inhibits the Cx43 interaction with drebrin, this would not be the mechanism [23]. The possibility exists that regulation from the β-catenin perspective inhibits the interaction with Cx43. Consistent with this is that: (1) the phosphorylation of β-catenin at S552 by protein kinase B increases the association between β-catenin and 14-3-3ζ, leading to β-catenin translocation into the cytosol and nucleus [65]; (2) the phosphorylation of β-catenin by casein kinase 1 is necessary for subsequent glycogen synthase kinase-3 phosphorylation and then degradation [66]; (3) the protein kinase A phosphorylation of β-catenin leads to the nuclear localization of β-catenin [67]. Interestingly, protein kinase B, casein kinase 1, and protein kinase A also phosphorylate Cx43 to promote synthesis, trafficking to the gap junction plaque, and channel opening.

The importance of identifying if and where a direct protein interaction occurs is in relationship to the phosphorylation of Cx43, because phosphorylation modifies the binding affinities of the Cx43 protein partners that regulate assembly, disassembly, and channel function [68]. For example, we demonstrated that mitogen-activated protein kinase phosphorylation of Cx43 increases the binding affinity for the E3 ubiquitin ligase neural precursor cell expressed, developmentally down-regulated 4 [69], which leads to Cx43 degradation [70,71]. Therefore, combining the location of a protein partner interaction on Cx43 along with the phosphorylation pattern under different homeostatic and pathological conditions will be crucial information for any potential therapeutic intervention. Here, we identified that β-catenin directly interacts with the Cx43CT domain, and that this interaction would be inhibited by Src phosphorylation of Cx43CT residues Y265 and Y313.

## 4. Material and Methods

### 4.1. Expression and Purification of Recombinant Proteins

The rat Cx43CT (V236–I382) (or (S255–I382) for the SPR study) polypeptide (unlabeled or ^15^N-labeled) cloned into the bacterial expression vector pGEX-6P-2 (GST-tagged; Amersham Biosciences, Little Chalfont, UK) was expressed and purified in 1× phosphate-buffered saline (PBS), as previously described [72,73]. Y313D and Y265,313D mutations in the Cx43CT plasmid were incorporated using the Quick Change Lightning kit (Qiagen, Hilden, Germany). Human β-catenin in pET-28a (+) was purchased from Addgene, expressed (unlabeled), and purified by Nickel affinity column (Buffer A: 50 mM Tris, 150 mM NaCl, 10 mM Imidazole, 2 mM β-mercaptoethanol (BME), pH 8.0; Buffer B: 50 mM Tris, 150 mM NaCl, 600 mM Imidazole, 2 mM BME, pH 8.0) followed by Anion exchange (Buffer A: 50 mM Tris, 2 mM BME, pH 8; Buffer B: 50 mM Tris, 1 M NaCl, 2 mM BME, pH 8). β-catenin ΔCT (M1-S680) was obtained by introducing a stop codon after serine 680 using the Quick Change Lightning kit (Agilent, Santa Clara, CA, USA). Purification was identical to the full-length β-catenin. β-catenin CT (S681–L781) was subcloned into the pET-16b vector, expressed (unlabeled), and purified by Nickel affinity column similarly to the full-length β-catenin. Purity and analysis for degradation was assessed by SDS-PAGE, and all of the polypeptides were equilibrated by dialysis in Slide-A-Lyzer G2 Dialysis Cassettes (Thermo Scientific, Waltham, MA, USA) in 1× PBS at pH 7.8 in presence of 2 mM dithiothreitol.

### 4.2. Nuclear Magnetic Resonance (NMR)

NMR data were acquired at 7 °C using a 600-MHz Varian INOVA spectrometer (Agilent, Palo Alto, CA, USA) upgraded with a Bruker Avance-III HD console (Bruker, Billerica, MA, USA) and outfitted with a Bruker z-axis PFG “inverse” triple-resonance cryogenic (cold) probe (Bruker). Gradient-enhanced two-dimensional ^15^N-HSQC experiments were used to obtain the binding isotherms of the ^15^N-labeled Cx43CT WT, Y313D, and Y265,313D at a constant concentration (35 μM) in the absence or presence of increasing amounts (up to 285 μM) of β-catenin, β-catenin CT, or β-catenin ΔCT. Data acquisition, processing, and analysis, including calculation of the dissociation constants (*K_D_*), have been previously described [14,46,69].

### 4.3. Circular dichroism (CD)

The CD experiment was performed on a JASCO J-815 CD spectrometer (JASCO, Mary’s Court, Easton, MD, USA) at 7 °C in the far UV (260–190 nm). Spectra of the β-catenin CT were collected in 1× PBS at pH 7.4, with a 0.1-mm path length quartz cell, using a bandwidth of 1 nm, an integration time of 1 s, and a scan rate of 50 nm/min. The final spectrum was obtained from the average of five scans. All of the spectra were corrected by subtracting the solvent spectrum acquired under identical conditions. CD data were processed and converted to mean residue ellipticity using Spectra Analysis from the Jasco Spectra Manager software, Version 2.05.01 (JASCO, Mary’s Court, Easton, MD, USA).

### 4.4. Surface plasmon resonance (SPR)

The SPR experiments were performed on a Biacore (GE Healthcare) 1000 at 25 °C. The Cx43CT (S255–I382) was immobilized onto a CM5 sensor chip by amine coupling, and the flow cell was equilibrated with the reaction buffer at a flow rate of 5 μL/min (213 mM phosphate buffer, pH 7.1). Then, 5 μL of either the β-catenin CT (4 μM), the Cx43EL1 (residues G38–R76, 10 μM, negative control), or the Cx43CT (10 μM, positive control) were injected over the chip, and the responses were recorded as resonance units (RU).
